# On-the-Fly Sleep Scoring Algorithm with Heart Rate, RR Intervals and Accelerometer as Input

**DOI:** 10.3390/s25072141

**Published:** 2025-03-28

**Authors:** Michele Guagnano, Sara Groppo, Luigi Pugliese, Massimo Violante

**Affiliations:** 1 Department of Control and Computer Engineering, Politecnico di Torino, 10129 Turin, Italy; sara.groppo@polito.it (S.G.); massimo.violante@polito.it (M.V.); 2 Sleep Advice Technologies, 10121 Turin, Italy; luigi.pugliese@satechnologies.eu

**Keywords:** wearable devices, smartwatch, sleep scoring, on-the-fly algorithm

## Abstract

In many applications, recognizing the depth of sleep (e.g., light, deep, REM sleep) while the subject is sleeping enables innovative features. For instance, in SAE Level 4 autonomous driving, a driver may need to takeover the vehicle control in case the autopilot is exiting its operational design domain. Depending on the depth of the sleep, the subject may need time to takeover effectively; hence, it is particularly relevant to know in which sleep stage the subject is (e.g., light sleep, deep sleep, and REM sleep), and possibly initiate actions to prevent the subject to remain in those sleep stages that lead to longer takeover time. Sleep stage classification can be achieved through an on-the-fly algorithm, which generates output in response to each input portion without knowledge of future inputs, unlike an off-Line algorithm that provides output just after receiving the entire input sequence. Various studies have analyzed algorithms or devices that identify sleep stages during the night; however, these typically require electroencephalography (EEG), which is obtrusive, or specialized devices. This study describes the development of an on-the-fly sleep-scoring algorithm using Heart Rate (HR), RR intervals, which is the distance between two consecutive heartbeats, and accelerometer data from a smartwatch, widespread, non-invasive, and affordable but accurate device. The subjects involved in our study wore a commercial off-the-shelf wearable device during a full night’s sleep, and were also monitored using a reference medical device to establish the ground truth by means of a full polysomnography (PSG) analysis. The on-the-fly sleep scoring algorithm based on smartwatch data was tested against PSG-based scoring, achieving 88.46% accuracy, 91.42% precision, and 93.52% sensitivity in sleep–wake identification. Deep sleep was correctly identified 69.38% of times, light sleep in 50.62%, REM sleep 62.02% and wakefulness 73.48% of times.

## 1. Introduction

The interest in the study of sleep is growing, thanks to awareness about the negative consequences of poor sleep quality. These can be daytime drowsiness, which can lead to low performance during work and to an increased risk of falling asleep while driving, weakening of the immune system, and generally lowering the quality of life [1,2].

First of all, quality sleep is characterized by sufficient Total Sleep Time (TST); specifically, 7 to 9 h are recommended. It must also be continuous, with few interruptions that are as short as possible. It must then be deep enough and consist of a short falling asleep time [3,4]. Finally, it is important to understand whether or not the individual in question is suffering from sleep disorders, such as respiratory disturbances, which could prevent sufficient energy recovery during the night and lead to daytime drowsiness [5].

The medical gold standard for the study of sleep is polysomnography (PSG), a medical examination in which electroencephalogram (EEG), electrocardiogram (ECG), electromyogram (EMG), electrooculogram (EOG), nasal flow, and movements during the night are monitored. Data collected are analyzed by a physician who does the sleep scoring and finds any sleep disorders [6,7,8].

Sleep scoring is the identification of the subject’s stage of sleep. During the night, light sleep (NREM1 and NREM2 phase), deep sleep (NREM3 phase), and REM sleep alternate. Light sleep is characterized by a slowing of cardiac and respiratory activity. Deep sleep consists of minimal Heart Rate (HR) and slow brain waves. REM sleep is distinguished by rapid eye movements (REM is, in fact, an acronym for Rapid Eye Movements) and by a higher HR [9,10]. The advantage of polysomnography is the ability to analyze several physiological parameters, including EEG, in order to obtain the most accurate scoring and reporting of disturbances. However, the disadvantages of this procedure are cost and invasiveness. A less invasive technique used to monitor sleep is actigraphy, which involves scoring through the study of movements detected by means of a wearable device [11,12]. Thanks to the improvements that wearable technology sensors have undergone, more and more devices have emerged that are capable of scoring using HR, Heart Rate Variability (HRV), and 3-axis acceleration [13,14]. Sleep is strongly related to the Autonomic Nervous System (ANS), which regulates many of the involuntary body functions, such as breathing, digestion, HR, and blood pressure. The ANS is divided into two main components: the sympathetic nervous system (which stimulates “fight or flight” responses) and the parasympathetic nervous system (which promotes rest and recovery). During sleep, the ANS plays a key role in regulating the different stages of sleep, especially in the transition between REM and non-REM sleep. HRV is a measure of the variation in time intervals between successive heartbeats (RR intervals), and it is closely related to the activity of the ANS [15]. Because of this relationship, wearable devices are able to perform sleep scoring quite accurately, although less accurate than a PSG, but cheaper and less invasive. In addition to RR intervals, there are several HRV metrics, such as SDNN (Standard Deviation of NN intervals) and RMSSD (Root Mean Square of Successive Differences), which are used in conjunction with HR and accelerometer to achieve a higher accuracy.

An important topic in the study of sleep is on-the-fly sleep scoring. An on-line (or on-the-fly) algorithm generates output in response to each input portion without knowing the future input [16]. An off-Line algorithm, instead, provides the output after having received the whole input sequence. In addition to being able to estimate how the previous night went, in some cases, it would be useful to be able to know at which stage the subject is at a specific moment. For example, sometimes it’s useful to avoid a subject falling into REM or deep sleep and to act instantaneously if it happens. Different studies have been conducted where the algorithms are able to identify the sleep stage during the night.

Thankachan et al. [17] developed an automated threshold calculation algorithm capable of identifying NREM, REM, and wake in mice. They used EEG and EMG and achieved an overall accuracy of 90%.

Koushik et al. [18] developed a time-distributed Convolutional Neural Network able to perform real-time sleep scoring. Their algorithm is able to identify five stages: N1, N2, N3, REM, and wake. They used a single-channel EEG obtained by a modified version of the Muse headband [19] and achieved an overall accuracy of 83.5%.

Rault et al. [20] developed another real-time algorithm able to detect sleep–wake patterns with a single-channel EEG with a resolution of 30s. They applied it to critically ill patients achieving a media sensitivity of 97.9% and a median specificity of 49.7% on the sleep–wake pattern identification.

Zhang et al. [21] implemented a real-time Automated Sleep Scoring device with one probe that senses blood oxygen, PPG, and actigraphy. It was able to perform four stages of sleep scoring with an accuracy of 75.1%.

Most of them require EEG, which is an invasive technology, or require ad-hoc devices. The idea behind this work is to implement an on-the-fly scoring algorithm that is able to work with signals available from a consumer smartwatch, in particular, HR, RR intervals, and 3-axis acceleration at 1Hz. The research significance of our concept is not just a chance to perform sleep scoring with HR, RR intervals, and accelerations, but the possibility of using these parameters in an on-the-fly sleep scoring algorithm. The obtainment of these data from cheap, low-invasive, and widespread wearable devices, can lead to interesting applications. A useful case for the on-the-fly sleep scoring algorithm could be while driving.

Despite the spread of autonomous driving, available systems are not completely autonomous. Cars with partial and conditional automation (L2–3) require human attention. The driver must be able to take back control of the vehicle when needed through the handover procedure [22]. In an automated context, drivers tend to be distracted and could become drowsy more easily, and drowsy driving is a public health problem that causes 17.6% of road accidents, according to an AAA Foundation study of the National Highway Traffic Safety Administration [23]. In particular, a deeper sleep state leads to a higher sleep inertia, preventing a timely control regain [24].

The problem with the proposed algorithm is the dependency from the 3-axis accelerometer, which could be strongly affected at the wheel, especially on rough terrains. So a second version of the algorithm which does not use the accelerometer was developed and tested.

## 2. Materials and Methods

Participants were monitored overnight in their homes through a full PSG. They signed informed consent before taking part in the data collection and filled out a form asking about gender, age, height, weight, and whether or not they were taking beta-blockers. Along with the PSG, they wore a smartwatch with a consumer-grade optical HR sensor. The used wearable exploits photopletysmography (PPG) to detect continuously HR at 1Hz, and the accelerometer sensor is able to provide 3-axis acceleration data up to 25Hz. Other than HR, the PPG sensor allows the detection of RR intervals. The used smartwatch was tested in different physical activity scenarios, achieving a Mean Absolute Percentage Error on Mean HR of around 3%. The previously mentioned data were extracted at 1Hz. PSGs were read by the doctor who produced a medical scoring.

### 2.1. Motion-Aware On-the-Fly Sleep Scoring Algorithm

Data acquired from the wearable device were passed to the Matlab algorithm capable of on-the-fly scoring (every second, the algorithm evaluates the sleep stage the subject is in based on the data acquired so far).

The algorithm takes HR, RR intervals, and a 3-axis accelerometer as input. Assuming that at the beginning of the recording the subject is awake, it starts looking for the physiological HR fall to find the sleep onset [9]. Next, it examines the various stages by following the known sleep stage behavior.

- LIGHT Sleep: NREM1 is a transitional phase between sleep and wakefulness, lasting a few minutes, in which the body begins to relax and from which it is easy to be awakened.
  Later, it transitions to NREM2, a phase more stable than NREM1 and with fewer movements that covers about 50% of total sleep. In the algorithm under consideration, NREM1 and NREM2 are combined to constitute the light sleep stage, a phase characterized by lower HR and Respiratory Rate than wakefulness.
- DEEP Sleep: NREM3 sleep, also known as deep sleep, is characterized by further lowering and regularization of HR and respiratory rate, movements are minimal. In adult individuals, it covers about 15–20% of total sleep and is mostly in the early part of sleep.
- REM Sleep: The last one is the Rapid Eye Movements (REM) phase, which owes its name to the irregular movements that the eyes make during this stage. It is characterized by brain activity similar to that during wakefulness, and consequently by higher cardiac activity than the other sleep phases. Total muscle atonia is present. It covers about 20–25% of total sleep and it is mostly in the final part of sleep [10,25].

The algorithm begins with the assumption that the user who starts it is awake. Even if the smartwatch requires more than 1 s to evaluate the different metrics, it starts evaluating them when worn, and their value is available when the application starts. Then, after each sample acquisition, if the subject is awake, the algorithm looks for an HR decrease and for the following HR stabilization inside the evaluation window. If it happens, the subject has fallen asleep. When the subject is sleepy, the algorithm behaves as follows:

- IF
  a large enough portion of HR is greater than the threshold AND
  a large enough portion of RR intervals is smaller than the threshold AND
  there are enough movements,
  THEN the subject is AWAKE;
- ELSE IF
  the standard deviation of the smoothed HR inside the window is smaller than the threshold,
  THEN the subject is in DEEP sleep;
- ELSE IF
  a large enough portion of the SDNN is larger than the threshold AND
  the movements are minimized,
  THEN the subject is in REM sleep;
- ELSE
  the subject is in LIGHT sleep.

**Figure 1:**
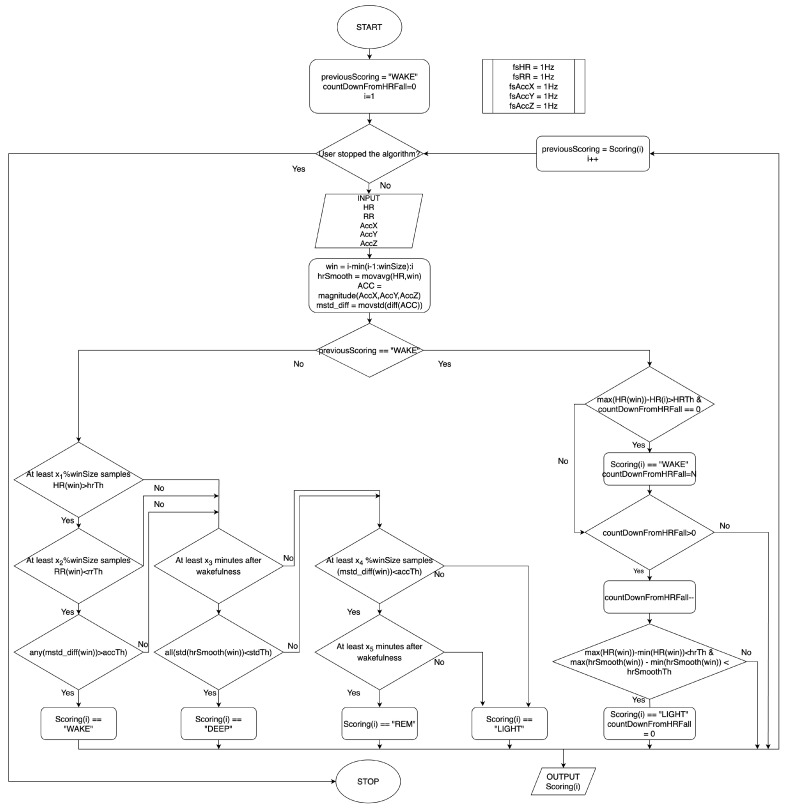
Flow chart of the motion-aware on-the-fly sleep scoring algorithm.

All the recordings were processed by this algorithm and the scoring obtained is compared with the PSG scoring written by the medical doctor. The flowchart in Figure 1 shows how the algorithm works according to the rules seen above. The 10 parameters (winSize, hrTh, rrTh, accTh, stdTh, ×1, ×2, ×3, ×4, and ×5) were defined based on a set of polysomnography tests other than those presented in this paper. In particular, HR, RR intervals, and accelerations behavior were observed in the different sleep stages. hrTh, rrTh, and accTh, are based on those metrics behavior during wakefulness, stdTh is based on how the standard deviation of the HR processed through a moving average decreased during deep sleep. The parameters ×3 and ×5 reflect a minimum time interval necessary for deep and rem stage transition. The tests presented in this paper are used to validate the performance of the algorithm by running a double-blind experiment: the PSG scoring is performed by the medical doctor independently from the algorithm results, and the algorithm results are collected without knowing the outcome of PSG scoring.

### 2.2. On-the-Fly Sleep Scoring Algorithm

As previously stated, a second version of the algorithm was developed, capable of performing on-the-fly sleep scoring without the accelerometer. The idea is similar to the previous one, where signals were compared to thresholds inside a window to identify the sleep stage. Also in this case, the algorithm begins with the assumption that the user who starts it is awake. Then, after each sample acquisition, if the subject is awake, the algorithm looks for an HR decrease and for the following HR stabilization inside the evaluation window. If it happens, the subject has fallen asleep. When the subject is sleepy, the algorithm behaves as follows:

- IF
  a large enough portion of HR is greater than the threshold AND
  a large enough portion of RR intervals is smaller than the threshold,
  THEN the subject is AWAKE;
- ELSE IF
  the standard deviation of the smoothed HR inside the window is smaller than the threshold,
  THEN the subject is in DEEP sleep;
- ELSE IF
  a large enough portion of the SDNN is larger than the 1st threshold AND
  a large enough portion of the SDNN is larger than the 2nd threshold,
  THEN the subject is in REM sleep;
- ELSE
  the subject is in LIGHT sleep.

**Figure 2:**
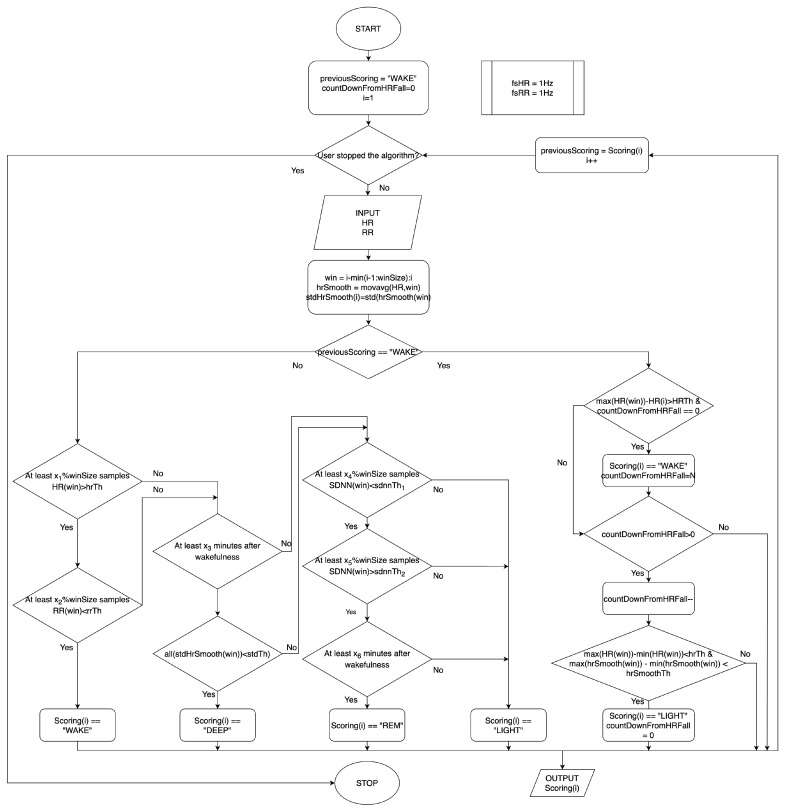
Flow chart of the on-the-fly sleep scoring algorithm.

The flowchart is shown in Figure 2.

## 3. Results

In this section, the results are reported to assess the performance of the developed algorithms. Two data sets were considered: one composed of 6 subjects recruited for overnight sleep monitoring specifically for the sake of this paper, and 20 subjects belonging to a public data set, the DREAMS Subject Database. The six subjects recruited for this study were monitored in their homes overnight with a PSG while wearing a smartwatch. The PSG data were read by a doctor who produced a scoring, which was used as ground truth for the study. Data acquired from the smartwatch, on the other hand, were sent as input to the algorithm and were used to generate a scoring to compare with the doctor’s scoring.

### 3.1. Motion-Aware On-the-Fly Sleep Scoring Algorithm

**Figure 3:**
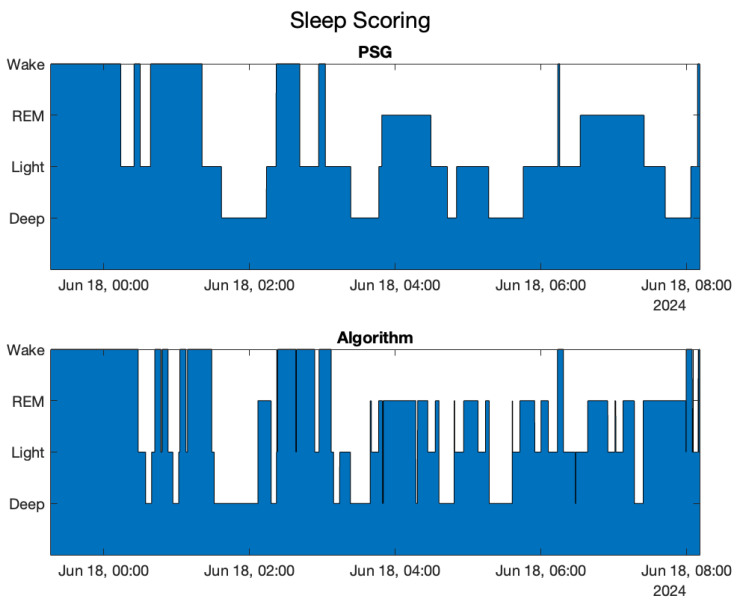
Medical sleep scoring and motion-aware on-the-fly algorithm sleep scoring compared for the 1st subject.

The following results were obtained: For each night session, the medical and the algorithm scoring overlapped second by second, as shown in the example in Figure 3.

In this paper, all figures in which medical sleep scoring and algorithm sleep scoring are compared refer to the same subject, the first subject. This decision was made in order to compare the same scoring representative of the data collected in the experimentation with the different algorithms shown. Table 1 shows, for each stage, how many times (in percentage) the stage was recognized as another phase. The main diagonal (in cyan) represents the percentage of successful identification for each stage.

The algorithm was evaluated also in terms of the sleep–wake pattern, taking into account the difference between Sleep Onset time, Awake time, Total Sleep Time (TST), and Wakeup After Sleep Onset (WASO). Results are shown in Table 2. Sleep Onset time and awakening time were compared for all the subjects, it was calculated how far ahead of medical scoring the times obtained with the algorithm were, and all values were averaged. The same was performed with TST and WASO.

Finally, the confusion matrix was performed also for the sleep–wake pattern, finding the following results:

Sensitivity=93.52%, Precision=91.42%, Accuracy=88.46%

### 3.2. On-the-Fly Sleep Scoring Algorithm

**Figure 4:**
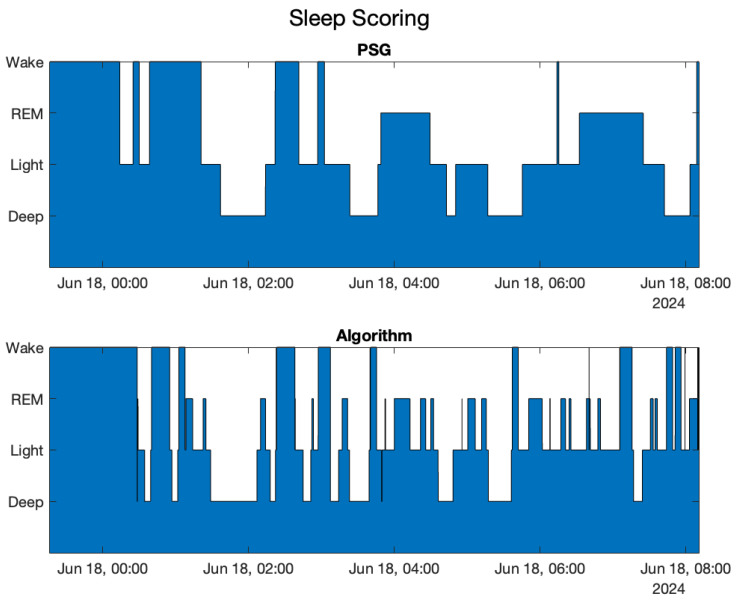
sleep scoring and on-the-fly algorithm sleep scoring compared for the first subject.

The same comparisons were performed for the algorithm without the accelerometer. Figure 4 shows the sleep scoring obtained by the algorithm compared with the one obtained by the medical doctor, Table 3 is the confusion matrix, and Table 4 analyzes the sleep–wake parameters.

By comparing the results of the motion-aware algorithm with those reported in this section, it is evident that REM phase identification is the one that suffers the most from the lack of accelerometer information. Conversely, the identification of the deep state is slightly affected. Finally, the confusion matrix was performed also for the sleep–wake pattern, providing the following results:

Sensitivity=91.64%, Precision=88.42%, Accuracy=85.94%

The paired T-test was performed to examine whether the motion-aware and the non-motion-aware algorithms have significative differences in terms of sleep stage classification metrics. Table 5 was made by comparing the results obtained with the two algorithms on each test, showing no significant difference between the two algorithms in terms of classification metrics. For precision evaluation, the Wilcoxon test was used instead of the *t*-test because it does not follow a normal distribution.

## 4. Commercial Off-the-Shelf Wearable Device and PSG Comparison

**Figure 5:**
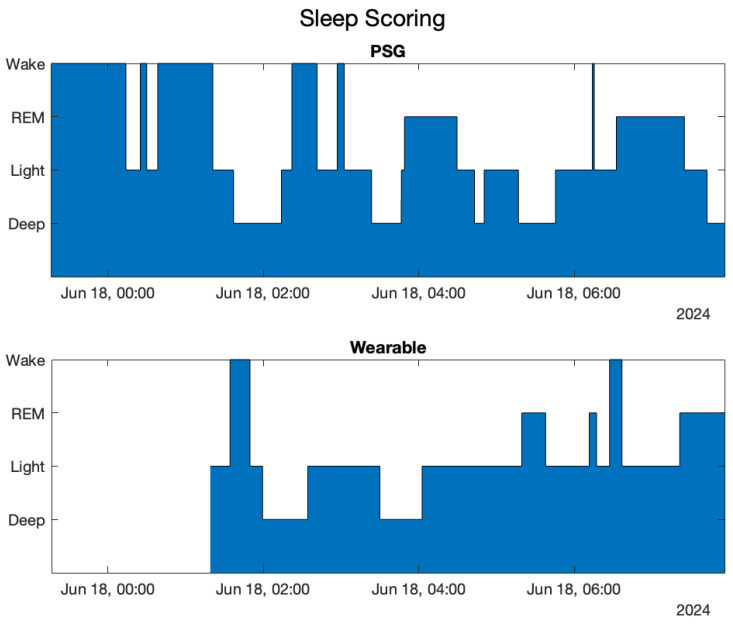
Medical sleep scoring and wearable sleep scoring compared for the first subject.

Since the monitored subjects were wearing the smartwatch, the scoring estimated by the device was also collected and compared with PSG scoring. The sleep scoring calculated by the wearable, shown in Figure 5, was taken from its application. The plot started when the sleep onset was detected, so the white portion, in the beginning, must be considered as ”Wake”.

Table 6 and Table 7 show, respectively, the confusion matrix and the sleep–wake parameters performances.

Finally, a comparison was made in terms of stage latency identification between the on-the-fly sleep scoring algorithm, the motion-aware on-the-fly sleep scoring algorithm, and the wearable device. For each stage, the average delay required for the algorithm to recognize the stage was calculated. The results are shown in Table 8.

## 5. On-the-Fly Sleep Scoring Algorithm Evaluation Using Public Data Set

The purpose of this section is to assess the performance of the presented algorithm using publicly available data set. For this purpose the DREAMS Subjects Database, available at https://zenodo.org/records/2650142#.XxVUU5NKjJ8 (accessed on 2 December 2024), was used. Given the characteristics of the available data set, the on-the-fly algorithm was used as information about motion of subjects as recorded by accelerometers was not available. From the dataset, that contains 20 full night polysomnographies, HR and RR intervals extracted from the Electrocardiograph (ECG) were used to estimate sleep stages with the on-the-fly sleep scoring algorithm, and results were compared with the medical scoring provided in the data set. The results are shown in Table 9.

**Figure 6:**
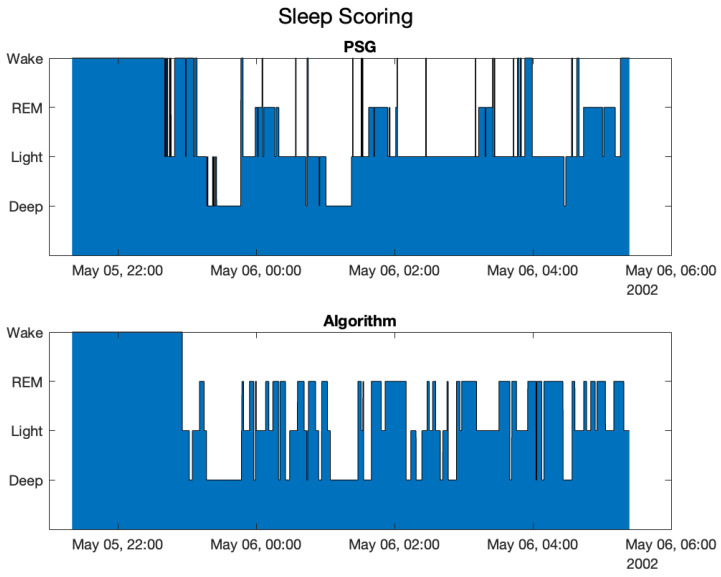
Medical sleep scoring and algorithm sleep scoring compared for a subject on the online dataset.

The results reported in the confusion matrix are comparable with those obtained with the six subjects analyzed previously. It is important to note that the algorithm uses a set of calibration parameters that are intended to balance the accuracy for the classification of all stages of sleep. In the use case of automated driving, the classification problem can be simplified in a problem to discriminate between non-deep sleep (where no action is needed) and deep sleep where an action is needed to bring the subject out of the deep sleep state. The calibration parameters can be optimized for such a use-case, thus improving the accuracy. A sleep scoring comparison is shown in Figure 6.

Table 10 shows the sleep–wake parameters performance.

Finally, the confusion matrix was performed also for the sleep–wake pattern, finding the following results:

Sensitivity=86.08%, Precision=84.27%, Accuracy=75.62%

## 6. Discussion

PSG is the gold standard for the study of sleep structure and pathology. It consists of monitoring a patient for one night by collecting data from different kinds of sensors, such as EEG and EOG. The next day, the collected data are read by a physician. This test allows the highest reliability in sleep detection, but it is invasive and expensive [26]. To fix these problems, there has been a growing interest in wearable devices such as smartwatches and smart rings, whose sensors have improved greatly in recent years and which enable remarkable results in sleep estimation. The problem addressed in this work is to create an algorithm for on-the-fly scoring, but one that can be used with wearable devices. Then, data were collected from wearable devices, and a Matlab algorithm was written to take input from the collected data and calculate the scoring. PSG was used as ground truth, as it is the most accurate technology for sleep scoring. A comparison of the results was made, and the following was determined.

The most difficult phase to identify was light sleep, which is a transitional phase, and has characteristics in common with wakefulness and REM stage, and lends itself to being confused with them. The easiest to identify were wakefulness and deep sleep. Wakefulness has HR and acceleration larger than every sleep stage, so it is usually more recognizable than the other stages, except for motionless wakefulness. Having both the HR and accelerometer can help in distinguishing between motionless wake and sleepiness. Deep sleep is also recognizable thanks to the smaller HR variation that characterizes it. The greater reliability in wakefulness recognition allowed the sleep–wake pattern values, namely TST, WASO, Sleep Onset time, and awakening time, to be more accurate. In particular, the Sleep Onset was shown to be the best identified parameter by looking for the physiological fall of HR. In the following works, the authors compared the sleep scoring performed by different wearables with the one obtained by PSG. Kinec et al. [27] compared five commercial sleep-tracking devices with actigraphy and PSG. Lee et al. [28] made a comparison between 11 commercial sleep devices, including wearable, nearable, and airable devices. Across all the devices, they found a bias toward the light sleep stage. In particular, speaking about wearables, they found that wakefulness is usually misclassified as light. Chinoy et al. [29] compared the performances of seven consumer sleep-tracking devices with the PSG, obtaining an overestimation of light sleep with respect to the medical device. Kim et al. [30] evaluated the sleep tracking ability of a specific smartwatch, the Samsung Galaxy Watch 3. They compared it to the PSG, obtaining weaker results on REM sleep. All of them agree on the overestimation on the Total Sleep Time (TST) at the expense of the Wakefulness After Sleep Onset (WASO). This is due to the difficulties in finding motionless wake, which sometimes causes our algorithm to misclassify it as light sleep. The main limitations on the results, with respect to the PSG, are the absence of EEG, which is critical for sure sleep stage identification, and the fact that the algorithm is on-the-fly, so evaluations cannot be conducted on the whole signals, but are performed while signals are collected.

An interesting application for the on-the-fly sleep scoring algorithm could be the identification of a deep sleep state while driving to allow the driver to recover. The problem with the proposed algorithm is the dependency on the 3-axis accelerometer, which could be strongly affected at the wheel, especially on rough terrains. So a second version of the algorithm which does not use the accelerometer was developed and tested. Despite the main application of the algorithm at the wheel was to detect a deep sleep state, all four stages were considered in the analysis for a more general use of the algorithm. The easiest to identify were wakefulness and deep sleep. This code provided the same results as the previous in deep sleep identification, since this stage was identified just using HR also in the previous algorithm. Performances in wakefulness and REM identification have slightly worsened because the contribution of the accelerometer is now missing. The performances on sleep–wake parameters, with the exception of the Sleep Onset Advance which depends just on HR, are also slightly worsened due to the lack of accelerations.

In other applications, such as sports rehabilitation, healthcare, and lifestyle monitoring, where the accelerations are not strongly affected by the environment, the motion-aware algorithm could be employed, providing better results. Movements are very helpful in recognizing wakefulness, usually characterized by higher accelerations, and REM sleep, which is known for its muscular atonia.

## 7. Conclusions

This work shows that an on-the-fly sleep scoring algorithm is possible also with HR and RR intervals. For better results, if the situation allows, it is possible to consider also the acceleration for sleep staging. This study demonstrates that an on-the-fly sleep scoring algorithm can be effectively implemented using Heart Rate (HR) and RR intervals data from consumer wearable devices, with acceptable accuracy and reliability for practical applications. The algorithm was tested with and without accelerometer input to assess the influence of motion data on sleep stage identification.

The results suggest that while accelerometer data improves the identification of certain stages, particularly wakefulness and REM sleep, a reliable level of performance is still achievable with HR and RR intervals alone.

The findings underscore the potential of such algorithms in applications where on-the-fly monitoring is essential, such as in safety-critical environments like autonomous driving. In these cases, the ability to exclude the accelerometer makes the algorithm less sensitive to external motion interference, offering a versatile solution. Future work could focus on further refining this model to enhance accuracy across all sleep stages, potentially incorporating additional physiological markers as wearable technology evolves.

## Figures and Tables

**Table 1:** Confusion matrix for sleep stage prediction with highlighted diagonal.

	True Deep	True Light	True REM	True Wake
Predicted Deep	69.38%	14.79%	7.56%	4.61%
Predicted Light	12.13%	50.62%	28.31%	19.88%
Predicted REM	15.90%	18.27%	62.02%	2.03%
Predicted Wake	2.59%	16.32%	2.11%	73.48%

**Table 2:** How sleep–wake parameters identified by the Motion-Aware On-the-Fly Sleep Scoring Algorithm are different from the ones found by the medical doctor.

Sleep Onset Advance	Wake Up Advance	TST Difference	WASO Difference
03 m 37 s	−08 m 46 s	10 m 25 s	14 m 46 s

**Table 3:** Confusion matrix for sleep stages.

	True Deep	True Light	True REM	True Wake
Predicted Deep	68.51%	15.44%	6.78%	4.21%
Predicted Light	19.36%	47.68%	38.50%	25.48%
Predicted REM	7.85%	24.02%	50.72%	7.49%
Predicted Wake	4.28%	12.86%	4.01%	62.82%

**Table 4:** How sleep–wake parameters identified by the On-the-Fly Sleep Scoring Algorithm are different from the ones found by the medical doctor.

Sleep Onset Advance	Wake Up Advance	TST Difference	WASO Difference
03 m 37 s	−10 m 41 s	18 m 58 s	15 m 14 s

**Table 5:** Statistical test results comparing the two sleep scoring algorithms.

Metric	Test Used	t-Statistic/W-Value	*p*-Value
Accuracy	Paired T-test	6.0908	0.5703
Sensitivity	Paired T-test	1.3582	0.2325
Precision	Wilcoxon	6.0000	0.4375

**Table 6:** Confusion matrix for sleep stages estimated by the wearable.

	True Deep	True Light	True REM	True Wake
Predicted Deep	44.73%	17.42%	7.54%	12.20%
Predicted Light	35.03%	60.41%	52.28%	70.52%
Predicted REM	14.15%	17.71%	33.88%	2.67%
Predicted Wake	6.09%	4.47%	6.30%	14.61%

**Table 7:** How sleep–wake parameters identified by the wearable are different from the ones found by the medical doctor.

Sleep Onset Advance	Wake Up Advance	TST Difference	WASO Difference
−30 m 16 s	08 m 30 s	41 m 19 s	20 m 00 s

**Table 8:** Sleep stage latency identification ($Time_{Medical}-Time_{Algorithm})$.

Sleep Stage	On-the-Fly Algorithm Latency	Motion-Aware On-the-Fly Algorithm Latency	Wearable Latency
Light	−00:06:26	−00:07:28	−00:30:16
Deep	00:03:25	00:01:39	−00:19:14
REM	00:03:06	−00:03:26	−00:35:21
Wake	−00:02:57	00:00:53	00:16:29

**Table 9:** Confusion matrix for the dataset online.

	True Deep	True Light	True REM	True Wake
Predicted Deep	58.40%	26.69%	11.93%	8.75%
Predicted Light	8.13%	23.43%	30.43%	20.11%
Predicted REM	9.97%	28.21%	43.02%	15.17%
Predicted Wake	23.50%	21.68%	14.62%	55.96%

**Table 10:** How sleep–wake parameters identified by the algorithm are different from the ones found by the medical doctor on the online scoring.

Sleep Onset Advance	Wake Up Advance	TST Difference	WASO Difference
−11 m 39 s	−14 m 03 s	07 m 00 s	04 m 15 s

## Data Availability

Restrictions apply to the availability of the first set of data. The second set of data is available in The DREAMS Databases and Assessment Algorithm at https://zenodo.org/records/2650142#.XxVUU5NKjJ8.
